# Inferring glaucoma status from prescriptions, diagnoses, and operations data: A Danish nationwide study

**DOI:** 10.1371/journal.pone.0292439

**Published:** 2023-12-06

**Authors:** Anna Horwitz, Marc Klemp, Jens Rovelt, Henrik Horwitz, Christian Torp-Pedersen, Miriam Kolko

**Affiliations:** 1 Department of Ophthalmology, Copenhagen University Hospital, Rigshospitalet, Glostrup, Denmark; 2 Department of Drug Design and Pharmacology, University of Copenhagen, Copenhagen, Denmark; 3 Department of Economics, University of Copenhagen, Copenhagen, Denmark; 4 Population Studies & Training Center, Brown University, Providence, Rhode Island, United States of America; 5 Department of Clinical Pharmacology, Bispebjerg and Frederiksberg Hospital, University of Copenhagen, Copenhagen, Nordvest, Denmark; 6 Department of Health, Science and Technology, Aalborg University Hospital, Aalborg, Denmark; New York University Grossman School of Medicine, UNITED STATES

## Abstract

**Purpose:**

To assess a new method for inferring glaucoma status using prescriptions data.

**Methods:**

The study population comprised all individuals living in Denmark in the period 1995 to 2018 and included 6,930,571 individuals. We used information from The National Prescription Registry on claimed prescriptions as the basis for our study (*N* = 223,592). We inferred glaucoma status using data on claimed prescriptions, in-hospital ICD-10 diagnoses, and in-hospital glaucoma surgeries. We infer glaucoma status in three ways using the prescription pattern: glaucoma inferred by (i) the use of a first claimed prescription, (ii) the use of a second claimed prescription with a gap of at least 90 days, and (iii) the use of a third claimed prescription for glaucoma medication, again with a gap of at least 90 days between prescriptions. Furthermore, we compared the results with alternative indications for glaucoma, namely in-hospital ICD-10-diagnosed glaucoma and in-hospital glaucoma surgery.

**Results:**

We first determined that glaucoma status could be inferred from claimed prescription data and found that a single claimed prescription was highly correlated with the more restricted composite measure of glaucoma (*R*^2^ = 0.80, *p* <0.0001), with a kappa coefficient of 80%. Focusing on individuals with a confirmed in-hospital glaucoma diagnosis, we found a high sensitivity of 88% using anti-glaucomatous prescriptions as a surrogate marker for primary open-angle glaucoma (POAG). We then derived several descriptive insights. The prevalence of glaucoma increased during the period from 1996 to 2018, while the incidence was constant. We also found a decreasing trend in the ratio of the number of people diagnosed annually in hospitals to the number of people filling prescriptions. This indicated a relative increase in the number of patients treated or managed in the secondary sector. Finally, using data on diagnoses and claimed prescriptions, we found that the proportion of total noncompliant patients, i.e., patients who do not claim their prescription at any time in the study period (two decades) was at most 11.8%. This share is calculated on the basis of diagnosed patients who did not have surgery. The results was not sensitive to the glaucoma inference rule.

**Conclusion:**

Anti-glaucomatous medicine prescriptions can be used to infer glaucoma status, with useful implications for epidemiological research. The sensitivity is particularly high for primary open-angle glaucoma (POAG).

## 1 Introduction

Glaucoma is a growing global health challenge and the leading cause of irreversible blindness worldwide [1]. It is an optic neuropathy characterized by the progressive degeneration of retinal ganglion cells and their axons, leading to visual disability. Currently, the pathogenesis of glaucoma is not fully understood, and strategies for prevention and disease management continue to challenge clinicians and the scientific communities.

Primary open-angle glaucoma (POAG) is the most common glaucoma subtype, accounting for around 74% of all cases in the western world [2]. Several individual characteristics are associated with the risk of developing glaucoma. Specifically, age is a strong risk factor, and data suggest an increasing number of individuals suffering from glaucoma due to ageing populations [3]. It is estimated that while 76 million individuals globally were affected by glaucoma in 2020, a total of around 112 million individuals will be affected in 2040 [4]. Furthermore, studies indicate that gender can be another risk factor [1–7]. However, the underlying factors that explain these differences remain unclear. Epidemiology can be used as a tool to shed light on possible associations and for this purpose the Danish registries have shown very high data reliability [8]. Similar registers exist in other countries, such as the Finnish Prescription Register, which was established in 1994 [9]. In order to exploit information in these registers, it is important to derive methods to identify e.g., glaucoma patients. This information can, as in this study, be used epidemiologically.

In Denmark, the Register of Medicinal Product Statistics records all prescriptions issued in both the hospital and private ophthalmology sectors. ICD-10 Diagnoses coding is only available for hospitalized individuals, as it is not mandatory for private ophthalmologists. Denmark’s ophthalmology sector is split into two main areas: the practice sector and the hospital sector. Roughly half of the 330 total specialists (160) hold a provider number, indicating they are practicing ophthalmologists, meaning that they are running privately-owned ophthalmology clinics that are integrated into and funded by the Danish public healthcare system [10]. The remaining specialists work primarily in public hospitals, with some in private facilities. Most glaucoma patients are most likely treated by private ophthalmologists, as hospitalization is not always necessary. The Danish National Health Service provides universal tax-supported healthcare, granting free access to private ophthalmology and hospitals. Private ophthalmologists are a major part of the Danish healthcare system, issuing most prescriptions for glaucoma without requiring a visit to a hospital.

In this epidemiological study, we developed a new method to infer glaucoma status from register-based data, i.e., in-hospital ICD-10 diagnoses, number of claimed prescriptions for anti-glaucomatous drugs and in-hospital glaucoma surgery. Our team, along with others, has previously used prescription data to infer glaucoma status [11, 12]. This methodology has subsequently been adopted in the analysis of English data [13]. With the new and refined method, we determined general trends in glaucoma incidence in the Danish population over two decades.

The main purpose of this study was to propose and evaluate the new method for inferring glaucoma status, and to estimate incidence, prevalence, and national trends in glaucoma over a period of 21 years in the Danish population using this method.

## 2 Methods

### 2.1 Study population and Danish register data

The study population comprised all individuals living in Denmark in the period 1995 to 2018 and included 6,930,571 individuals. Our dataset contained health-related information registered on the individual. Individuals who migrated into or out of Denmark after 1995 were excluded. Information on in-hospital diagnoses originated from the Danish National Patient Register, which covers the medical history and other information on all hospitalizations since 1977. In this register, diagnoses were annotated according to the International Classification of Diseases–10 (ICD–10).

Data on claimed prescriptions were from the National Danish Registry of Medicinal Products. This register contains information from all primary and secondary sector pharmacies in Denmark. All pharmacies in Denmark are obliged by law to register all prescriptions using the social security number, the so-called CPR (“Det Centrale Personregister”) number. Therefore, our dataset provided comprehensive information on anti-glaucomatous drug proclamations.

### 2.2 Inferring glaucoma status

We constructed a composite measure consisting of three factors that indicated a valid glaucoma diagnosis. The first factor was based on recorded glaucoma diagnoses in the hospital, which we denote as “*D*”. The second was based on glaucoma surgery in the hospital, which we denote as “*O*”. The third was based on the number of claimed prescriptions for anti-glaucomatous drugs, which we denote as “*P*”. *D* and *O* were considered highly reliable because a glaucoma diagnosis and/or surgery were definite indicators of glaucoma. D, O, and P represent different shares of the population. *P* represents the largest share at 86.05% (158,242 individuals), followed by *D* at 40.32% (74,177 individuals), and *O* at 6.99% (12,852 individuals) out of the total of 183,885 individuals. These and other proportions are visually represented in a Venn diagram in S1 Fig, panel B of S1 Appendix. In addition, data on diagnosis included information about glaucoma subtypes e.g., POAG, primary angle-closure glaucoma (PACG), elevated intraocular pressure (IOP) and secondary glaucoma. Unfortunately, *D* and *O* only captured patient cases from the primary sector, and *P* could also include a prescription for anti-glaucomatous drugs used for other therapeutic indications such as increased postoperative IOP. To mitigate this problem, we adapted *P* to only include individuals with at least three claimed prescriptions. Incidence and prevalence rates were subsequently calculated using this composite measure.

**Fig 1:**
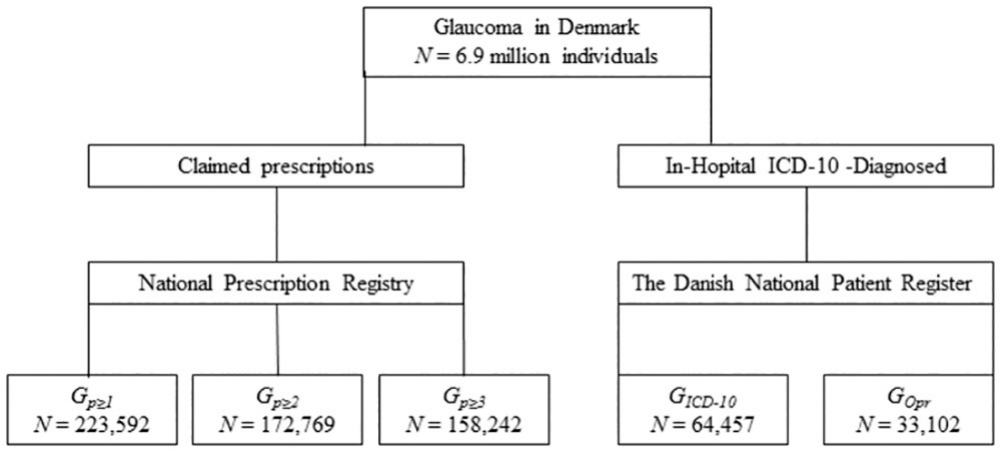
Flow chart. Flow chart of glaucoma cases in the Danish population in the period from 1996 to 2018 and the numbers entailed in each measure. The National Prescription Register and National Patient Register were used to identify all relevant individuals.

Each of the three measures is explained in more detail in the following subsections. A diagram of the relationship between the measures can be found in Fig 1.

#### 2.2.1 Glaucoma status inferred by in-hospital ICD-10 diagnoses (*D)*

We identified hospital-affiliated glaucoma patients in our dataset using ICD-10 codes, applicable to in-hospital patients only. The following IDC-10 codes were used. POAG: H401 (not H401F), PACG: H402, ocular hypertension (OHT): H400 (high IOP, but no glaucomatous optic neuropathy), other glaucoma: H403, 404, 405, 406, 408, and 409, and the diagnosis termed “glaucoma *hypotensivum*”: H401F.

Glaucoma is a chronic disease that, once diagnosed, requires lifelong treatment. Unlike certain other conditions, glaucoma does not have periods of relapse or remission. Therefore, in our analysis, we consider the initial diagnosis as a singular case, regardless of the course of the disease over time.

#### 2.2.2 Glaucoma status inferred by in-hospital surgery (*O)*

We also identified cases of glaucoma from information about in-hospital surgeries. Individuals who underwent either trabeculectomy/minimally invasive glaucoma surgery/tube shunt surgeries (KCHB) or laser trabeculoplasty (KCHD) were considered glaucoma patients. The incidence of these elements are shown in S2 Fig of S1 Appendix.

#### 2.2.3 Glaucoma status inferred by claimed prescriptions *(P)*

We used data from the National Danish Registry of Medicinal Products Statistics to find all individually claimed prescriptions for anti-glaucomatous medication. This dataset also included claimed prescriptions from out-hospital settings. Anatomical Therapeutic Chemical (ATC) codes, specifically S01E subcodes, were applied to identify the use of anti-glaucomatous medication. We defined the onset of glaucoma as the date of the first claimed prescription.

To examine the importance of the number of claimed prescriptions on the results, *P* was further subdivided:

- One claimed prescription (*P*_*≥1*_): the patient has redeemed at least one prescription for anti-glaucomatous medication.
- Two claimed prescriptions (*P*_*≥2*_): the patient has redeemed at least two prescriptions for anti-glaucomatous medication and has been treated for more than three months. Note that the set of patients captured by *P*_≥2_ ⊆ the set of patients captured by *P*_≥1_.
- Three claimed prescriptions (P_*≥3*_): the patient has redeemed at least three claimed prescriptions for anti-glaucomatous medication and has been treated for more than three months. Note that the set of patients captured by *P*_≥3_ ⊆ the set of patients captured by *P*_≥2_.

Since anti-glaucomatous drugs can in some cases be administered to a person without glaucoma, we mainly focus on *P*_*≥3*_. The overlaps between the *D*, *O* and *P*_*≥3*_ measures are shown in S1 Fig of S1 Appendix.

#### 2.2.4 Composite measure of glaucoma status

A composite measure of glaucoma status was defined using the presence of either an in-hospital glaucoma diagnosis *D*, and/or in-hospital glaucoma surgery (trabeculectomy/ minimally invasive glaucoma surgery (MIGS)/tube shunt surgery) *O*, and/or three claimed prescriptions *P*_*≥3*_. Denoting this measure by *G*_*c*_, we have $G_{c}=I_{D=1\vee O=1\vee P_{\geq 3}=1}$, where *I* is the indicator function. Patients were considered incident at the earliest date of either of the three measures.

### 2.3 Statistics

Incidence rates were calculated using *G*_*c*_. The incidence rate for a given year was defined as the number of new glaucoma cases divided by the size of the Danish population in the particular year. Incidence rates were also calculated for various sub-groups, e.g., for five-year age groups and males and females, using the number of new glaucoma cases in each group and the population size of each group. The prevalence was calculated as *G*_*c*_ divided by the size of the Danish population. Again, analogous measures were calculated for various subgroups.

We use linear regression and determine the statistical significance of the parameters through two-sided t-tests. We adhere to a significance level of 5% for these tests. All our statistical analyses are conducted using SAS 9.4.

Data from 1995 and 2018 may include information from previous years or be incomplete, thus the period 1996–2017 will be used in cases where this may affect estimates, as specified in the relevant text.

### 2.4 Ethics

The Danish Data Protection Agency approved the study (2007-58-0015, int. ref: GEH-2010-001). The data is anonymized. Retrospective register-based studies do not require ethical approval in Denmark.

## 3 Results

### 3.1 Assessment of glaucoma information sources from Danish register data

#### 3.1.1 Prescription patterns for glaucoma patients

**Fig 2:**
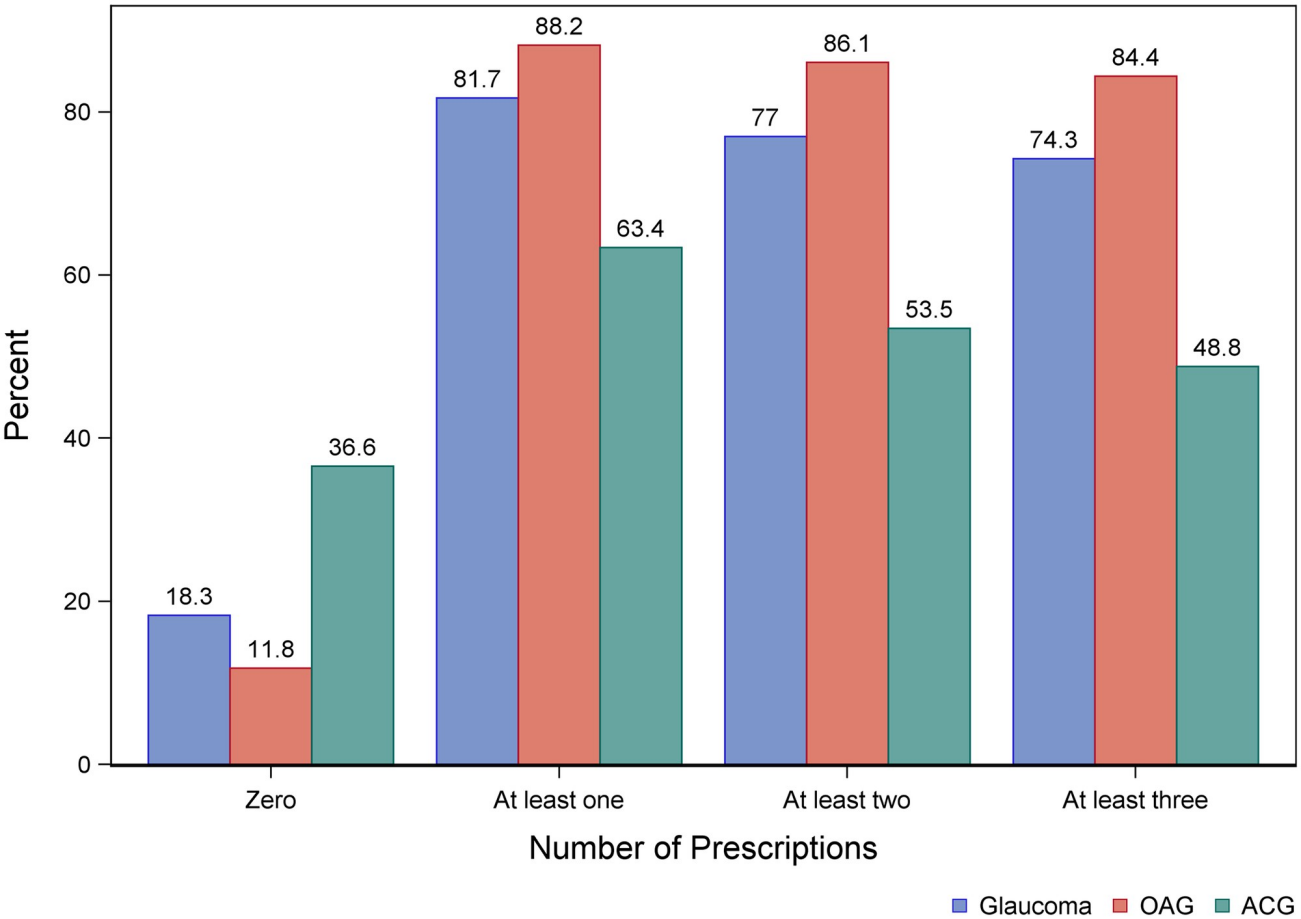
Frequency of prescriptions in patients diagnosed with glaucoma. The frequency of prescriptions for patients diagnosed with glaucoma who have not undergone surgery. *Blue*: Total proportions of prescriptions for both POAG and PACG. *Red*: Proportions of prescription for individuals with POAG. *Green*: Proportions of prescriptions for people with PACG.

We found that 81.7% of people diagnosed with glaucoma were identified by at least one prescription, 77.0% by at least two prescriptions, and 74.3% by at least three prescriptions for anti-glaucomatous drugs. Furthermore, we found that 18.3% of the unoperated glaucoma patients had not claimed any prescriptions (Fig 2). Moreover, we found that 89.4% of patients identified by the composite measure could be identified using the existence of at least one prescription.

As illustrated in S3 Fig of S1 Appendix, the sensitivity of claimed prescriptions among in-hospital ICD-10 diagnosed glaucoma patients was higher for individuals above 40 years of age as compared to younger individuals. Furthermore, glaucoma status inferred using the *P*_*≥3*_ criterion was more strongly correlated with POAG than PACG.

Our assessment of data sources indicated that claimed prescriptions served as an accurate marker of glaucoma status. Because prescription information was available for the entire population, our results suggested that glaucoma status can be approximated by the number of claimed anti-glaucomatous prescriptions. However, since some individuals may be diagnosed with glaucoma or undergo glaucoma surgery without claiming prescriptions for anti-glaucomatous medicine, our preferred glaucoma status measure was the composite measure *G*_*c*_.

#### 3.1.2 Assessing the validity of anti-glaucomatous prescription as an indicator of glaucoma status

We examined the *kappa* coefficient between our composite measure (*G*_c_) and the measure of glaucoma defined by redemption of at least one prescription for anti-glaucomatous medication (*P*_≥1_). This latter measure has been used in earlier research, but the validity of this measure has not been determined before. Bearing in mind that the magnitude of the *kappa* coefficient can be difficult to interpret, we found that *P*_≥1_ is a substantially good indicator for glaucoma status. The *kappa* coefficient is:

$$\kappa =\frac{p_{0}-p_{e}}{1-p_{e}}=80\%$$

where *p*_0_ is the observed proportionate agreement between *G*_c_ and *P*_≥1_ and *p*_*e*_ is the hypothetical probability of random agreement between *G*_c_ and *P*_≥1_.

We include 2-by-2 tables for the number of individuals identified by the composite measure and the three prescription-based measures in S1 Table of S1 Appendix. This table also reports the related Jaccard indices.

#### 3.1.3 Additional findings

We find, for the period 1996–2018, that the number of individuals captured by *P*_≥1_ is 223,592; the number of individuals captured by *P*_≥2_ is 172,769 (around 77% of *P*_≥1_), and the number of individuals captured by *P*_≥3_ is 158,242 (around 71% of *P*_≥1_ and 92% of *P*_≥2_). Furthermore, most glaucoma cases could be inferred from the *P*_*≥3*_ criterion alone followed by *D* and *O*. S1 Fig in S1 Appendix illustrates the overlap between each sub-measure.

The share of patients who have ben treated in hospital and thereby given an ICD10 diagnosis was 23.2% of patients satisfying the *P*_≥1_ criterion; 28,2% of patients satisfying the *P*_≥2_ criterion and 29,6% of patients satisfying the *P*_≥3_ criterion. The similarity coefficient is 0.22 between *P*_*≥1*_ and *D*, 0.26 between *P*_≥2_ and *D* and 0.27 between *P*_≥2_ and *D*.

The phi correlation between the various glaucoma data sources was assessed. The analysis revealed a high correlation between *P*_*≥1*_, *P*_*≥2*_, and *P*_*≥3*_ (*r* = 0.87–0.97; in all cases *p*<0.001). Furthermore, our analysis found that the *P*_*≥3*_-based measure was more strongly correlated with POAG (*r* = 0.38, *p*<0.001) than PACG (*r* = 0.13, *p*<0.001). Finally, our analysis showed that PACG had the highest correlation with IOP-lowering surgery, *O* (*r* = 0.46, *p*<0.001) (Table 1).

**Table 1:** Pearson correlation coefficients between various glaucoma status definitions.

**Phi coefficients** *N* = 6,930,571							
	*P*_*≥3*_	*P*_*≥2*_	*P*_*≥1*_	*D* (Any Glaucoma)	*D* (PACG)	D (POAG)	*O*
*G*_*c*_	0.93***	0.90***	0.80***	0.59***	0.28***	0.42***	0.36***
*P*_*≥3*_		0.96***	0.84***	0.46***	0.13***	0.39***	0.25***
*P*_*≥2*_			0.88***	0.45***	0.13***	0.38***	0.26***
*P*_*≥1*_				0.43***	0.14***	0.34***	0.25***
*D* (Any Glaucoma)					0.46***	0.71***	0.52***
*D* (PACG)						0.09***	0.45***
*D* (POAG)							0.32***

The table shows the phi coefficients for the various glaucoma status definitions (*N* = 6,930,571). It establishes high coefficients between measures of glaucoma status inferred from one, two or three claimed prescriptions of anti-glaucomatous medications in the study period (*r* = 0.80–96; in all cases *p*<0.001). Furthermore, it establishes that the *P*_*≥3*_-based measure is more strongly correlated with POAG than PACG. Finally, it shows that PACG has the highest correlation coefficient with IOP-lowering surgeries (*r* = 0.45, *p*<0.001).

*** p<0.01.

### 3.2 Trends and determinants of incidence and prevalence of glaucoma

#### 3.2.1 Overall incidence and prevalence

We investigated the patterns of incidence and the lower bound of prevalence of glaucoma, based on the *D*, *O* and *P* critera. We use 2017 data for annual statistics as the latest year’s data may be incomplete. Prevalence estimates are conservative due to data truncation at the start and end, excluding individuals who only met the *G*_*c*_ criterion before the first year and those who redeemed anti-glaucoma medication a third time only after the latest year.

Based on *G*_*c*_, the lower bound of the prevalence of glaucoma in Denmark in 2017 was 1.61%. Based on the prescription criteria, the lower bound of the prevalence ranged from 1.4% for *P*_*≥1*_ to 1.9% for *P*_*≥3*_ in the same year. The measure using at least one claimed prescription may indicate a higher glaucoma rate than the measure using at least three claimed prescriptions. This is due to the combined measure (*G*_*c*_) being based on a minimum of three claimed prescriptions to reduce false positives, hence *P*_*≥1*_ is likely greater than Gc.

**Fig 3:**
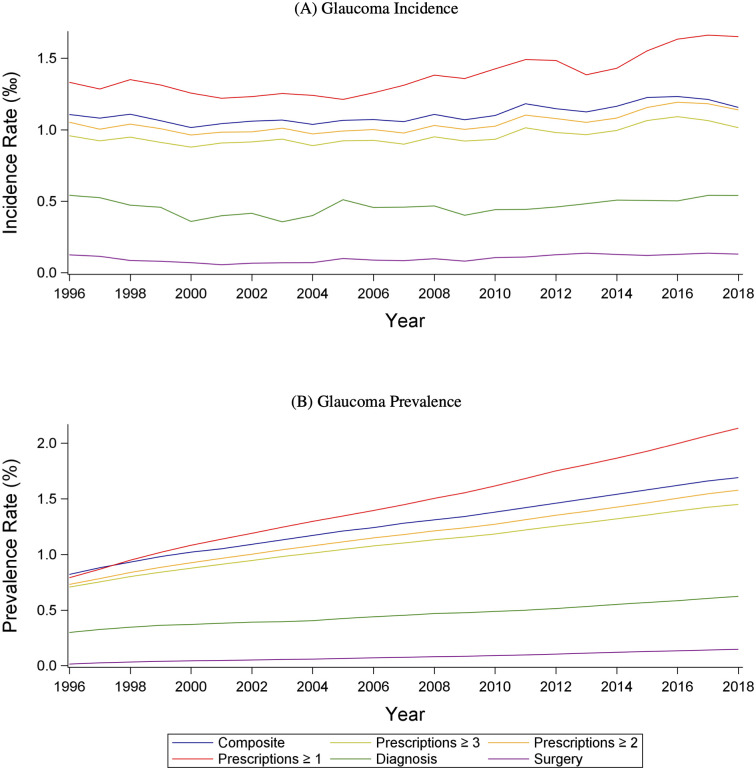
Incidence and prevalence of glaucoma for the various measures. “Composite” is *G*_*c*_, “Prescription>3” is *P*_*≥3*_, “Prescription>2” is *P*_*≥2*_, “Prescription>1” is *P*_*≥1*_, “Diagnosis” is the measure based on in-hospital ICD-10-coded diagnoses, and “Surgery” is the measure based on in-hospital surgery.

We have also applied our method and calculated trends over a 21-year period. Fig 3 depicts the development of incidence and prevalence. Panel A illustrates the total glaucoma incidence rate calculated with the different measures. The overall incidence based on *G*_*c*_ was rather constant throughout the period. The prevalence rates are depicted in Panel B. Overall, we demonstrated that incidence and the lower bound of the prevalence increased over the 21-year period.

Similar conclusions were reached using glaucoma subtype measures based on the diagnosis data. S2 Fig, Panel A in S1 Appendix, depicts the incidence of ICD-10-diagnosed glaucoma cases in the hospital over the 21-year period, divided into POAG, PACG, OHT, and secondary glaucoma. It reveals that POAG is the most frequent type of glaucoma in Denmark, and that there are similar trends in the prevalence’s of the different types of glaucoma. S2 Fig, Panel B in S1 Appendix, depicts the frequency of different glaucoma surgery over the 21-year period.

#### 3.2.2 Determinants

**Fig 4:**
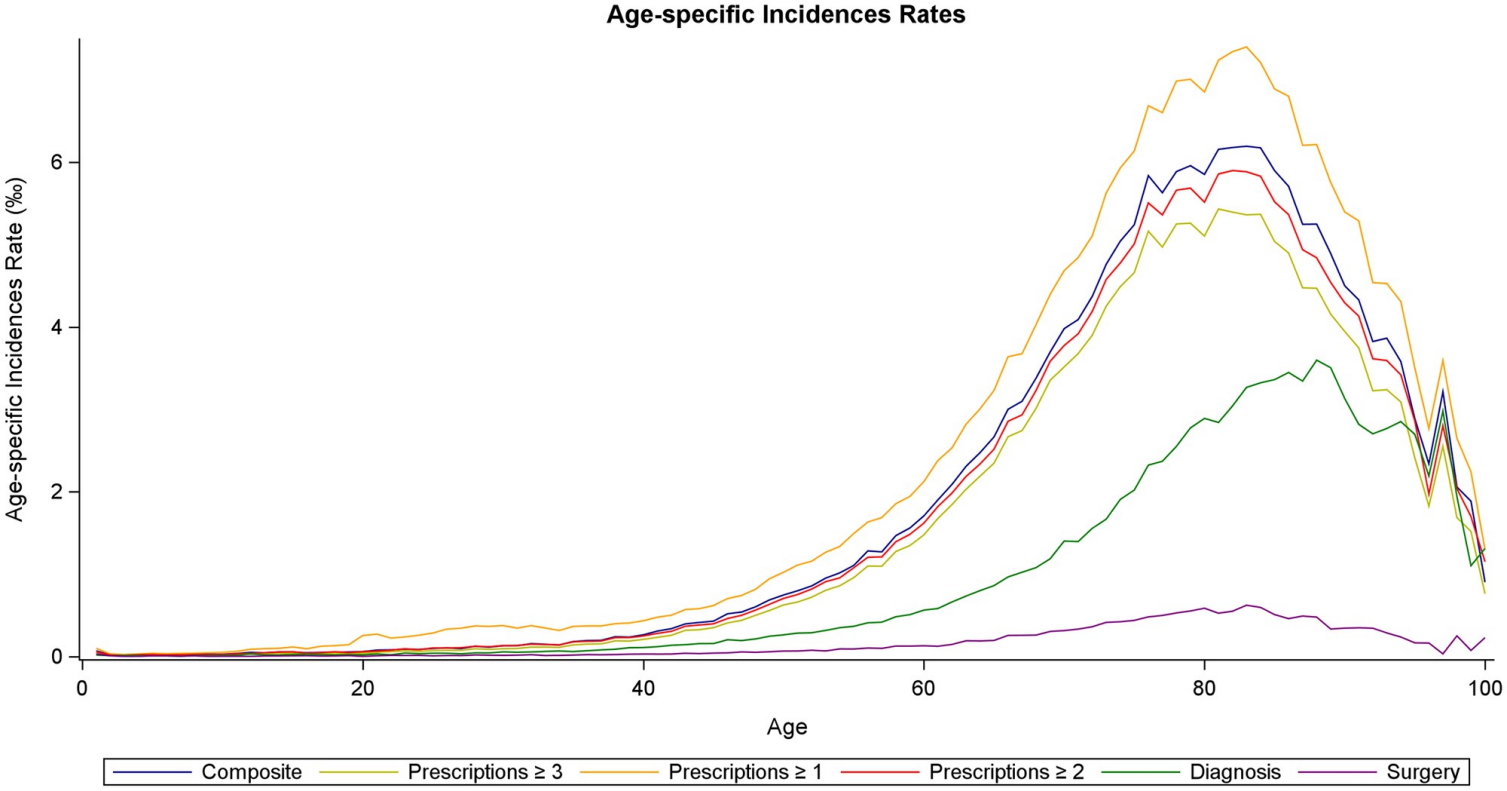
Age-specific glaucoma incidence rates. Incidence rate per 1000 person-years (where glaucoma is observed in the 21-year period) is shown for the claimed prescriptions sub-measures as well as the combined glaucoma measure.

We explored and visualized the association between age and the various indicators of glaucoma age. S4 to S6 Figs in S1 Appendix demonstrate similar age-related patterns for prescriptions, diagnoses and surgeries as well as the three sub-measures of prescription-based glaucoma status, indicating that the various measures capture the same age-related association, except for the magnitude. We also found approximately similar age-related associations when measuring incidence with each of the indicators (Fig 4). The major divergence between the measures (again, except for the magnitude) was that peak incidence occurred later based on the surgery data and later still using the diagnosis data compared to the prescriptions data.

#### 3.2.3 Other findings

**Fig 5:**
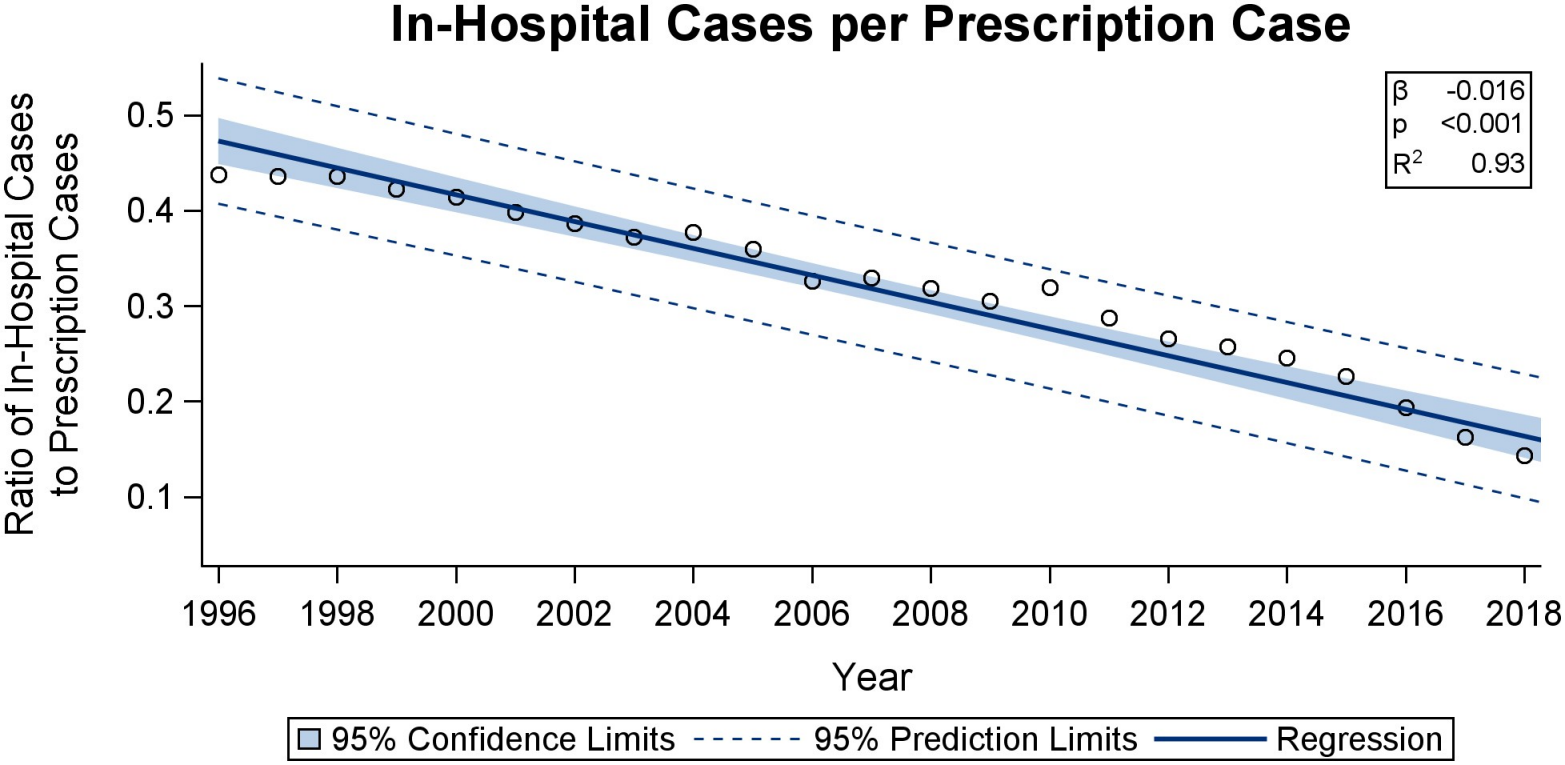
In-hospital cases per prescription case. The figure depicts the ratio between the number of patients with in-hospital diagnoses per claimed prescriptions meaning the ratio between primary and secondary diagnosis. It shows a significant decrease throughout the period from 1996 to 2018, suggesting that an increasing share of patients are diagnosed in the secondary sector. Solid lines represent the regression fit. Shaded areas represent the 95-% confidence limits. Dotted lines represent the 95-% prediction limits.

*Primary versus secondary sector*. We can use our data to get an indication of the relative development in glaucoma treatment and management between the primary and the secondary sectors. Since, as explained above, claimed prescriptions cover the entire population, whereas diagnoses are known only for the primary sector, the evolution of the ratio between the number of individuals prescribed anti-glaucomatous medicine and the number of individuals diagnosed with glaucoma is indicative of the relative size of treatment in the primary sector. This assumes a constant prescription-rate for in-hospital diagnosed individuals as well as a constant relative compliance rate between the two sectors. Fig 5 depicts the number of people diagnosed in hospital per individual with three or more claimed prescriptions. The Fig suggests a decreasing trend in the ratio between the number of people diagnosed annually in hospital relative to the number of people with claimed prescriptions. This indicates a relative rise in the number of patients treated or managed in the secondary sector.

*Total non-compliance*. We found that 11.8% of non-operated patients diagnosed in the hospital with POAG did not redeem any medication in the entire study period. Taken at face value, this implies that the upper bound on the share of total non-compliers is 11.8% provided that the clinicians have coded their diagnoses correctly according to ICD-10 classification. The corresponding upper bound for PACG is 36.6% and the overall upper bound is 18.3% (see Fig 2). Restricting the data to 1996 and 2017, respectively, yields upper bounds of 17.4% and 25.0%, indicating an increase in scope for non-compliance.

## 4 Discussion

### 4.1 General discussion

In this nationwide population-based study, we assessed and compared various ways to infer glaucoma status. To our knowledge, this is the first study to investigate and compare the validity of different register-based information to determine a person’s glaucoma status.

Previous studies have employed prescription data to infer glaucoma status in a variety of ways. A common approach is to infer a positive glaucoma diagnosis from at least one claimed prescription of anti-glaucomatous medication [11, 12, 14–18]. Other methods of using prescription data as a marker for glaucoma, such as focusing on the number of prescriptions per person, have also been utilized [13].

In this present study, we developed a composite measure for glaucoma based on all available and relevant information from the Danish registries: number of redeemed prescriptions, diagnosis codes and types of surgery. This composite measure was yielded a 15% higher incidence rate than the measure based on prescriptions alone. We showed that identifying patients based on the simple criterion of redeeming one or more anti-glaucomatous medication prescription (*P*_≥1_) yields a *kappa* coefficient of 80% with respect to the composite measure, indicating a substantial marker that is bordering the “almost perfect” strength-of-agreement category as defined in [19]. Our analysis suggested that prescriptions alone can serve as a useful and simple marker for glaucoma, and that the composite measure is a better measure that contains additional useful information.

Our study supports the notion that glaucoma is an increasing problem in the aging Danish population. The increased prevalence in Denmark can be compared with global findings. For example, a 2016 study predicted that the overall prevalence of POAG will increase by 0.1–0.2% from 2015 to 2020 due to the increase in average life expectancy [4]. However, it is worth bearing in mind that an increase over time can also be attributed, at least in part, to improved diagnostic methods, improved clinical disease knowledge and a possible change in initiation of medical treatment.

### 4.2 Strengths and limitations

#### 4.2.1 Data

Our data set had many strengths but also limitations. The primary strength of the data was that it covered the entire Danish population over a longer period. It contained individual-level information on glaucoma as well as a number of potentially important characteristics. Furthermore, the data was collected systematically and consistently. The collection of this type of data is regulated by law in Denmark [8].

The primary purpose of the administrative register data using ICD-10 codes from hospitals is to monitor hospitals and healthcare utilization. Data quality and completeness depend on the accuracy of the clinicians’ diagnoses and the accuracy of recording diagnoses in hospital registries. Of all the hospitalized patients with diagnoses or surgery information, we expected that the majority were registered in the National Patient Registry. However, a few patients could be missing, and we also anticipated that some patients will be misclassified (i.e., not classified correctly according to the gold standard).

Moreover, the ICD-10 coding scheme—relevant for the diagnosis data—is not reported from the secondary sector (i.e., private ophthalmologists). Likewise, no surgeries from the secondary sector were reported. This was the primary reason for combining diagnosis and surgery data with claimed prescription information.

Another source of underreporting in the data was latent cases i.e., persons that have not yet been diagnosed. To estimate the number of undiagnosed cases, a screening study would be necessary. The existence of latent cases may generally generate differences in estimates of incidence and prevalence in clinical screening studies and register-based studies. A final drawback of the present data is the lack of information on visually significant ‘end-organ damage’.

#### 4.2.2 Methods

A main strength of the study was the use of data from the entire Danish population, thus eliminating the potential for selection bias in the estimation of the overall incidence and prevalence. Furthermore, our analysis controlled for a range of potentially confounding factors and employed several estimators (OLS,) to calculate and investigate the robustness of the results concerning alternative estimators.

## 5 Conclusion

We found that information about prescriptions of glaucoma medication from the Danish registers is of great use in the inference of glaucoma status. Claimed prescriptions have a particularly high sensitivity for identifying POAG diagnoses.

## Supporting information

S1 Appendix Supporting figures and supporting table. (PDF)
