# Supplementary material for: Inferring glaucoma status from prescriptions, diagnoses, and operations data: A Danish nationwide study
Source: PLoS One. 2023 Dec 6;18(12):e0292439. doi: 10.1371/journal.pone.0292439 (PMC10699638; doi:10.1371/journal.pone.0292439)
Supplement: S1 Appendix — (PDF) [file pone.0292439.s001.pdf]

S1 Appendix

Inferring Glaucoma Status from Prescriptions, Diagnoses, and Operations Data
A Danish Nationwide Study

Anna Horwitz, Marc Klemp, Jens Rovelt, Henrik Horwitz, Christian Torp-Pedersen, Miriam Kolko

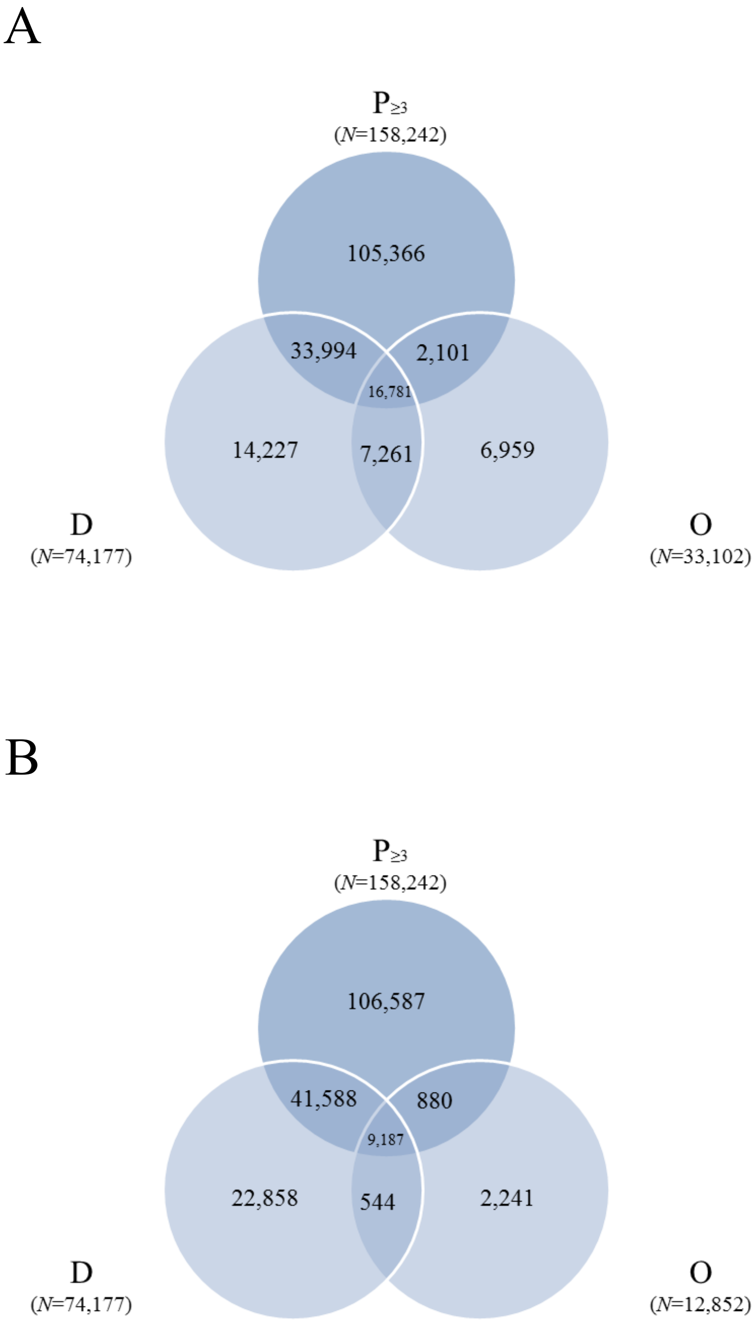

S1 Fig. Venn diagram. This diagram shows the number of glaucoma patients with either claimed prescriptions (P≥3), in-hospital ICD-10 glaucoma diagnosis (D), or IOP-lowering surgery (O). Panel A: All IOP-lowering surgery (O). Panel B: Individuals who underwent either trabeculectomy/ micro-invasive glaucoma surgery/tube shunt surgeries (KCHB) or laser trabeculoplasty (KCHD) were considered glaucoma patients. (O).

A

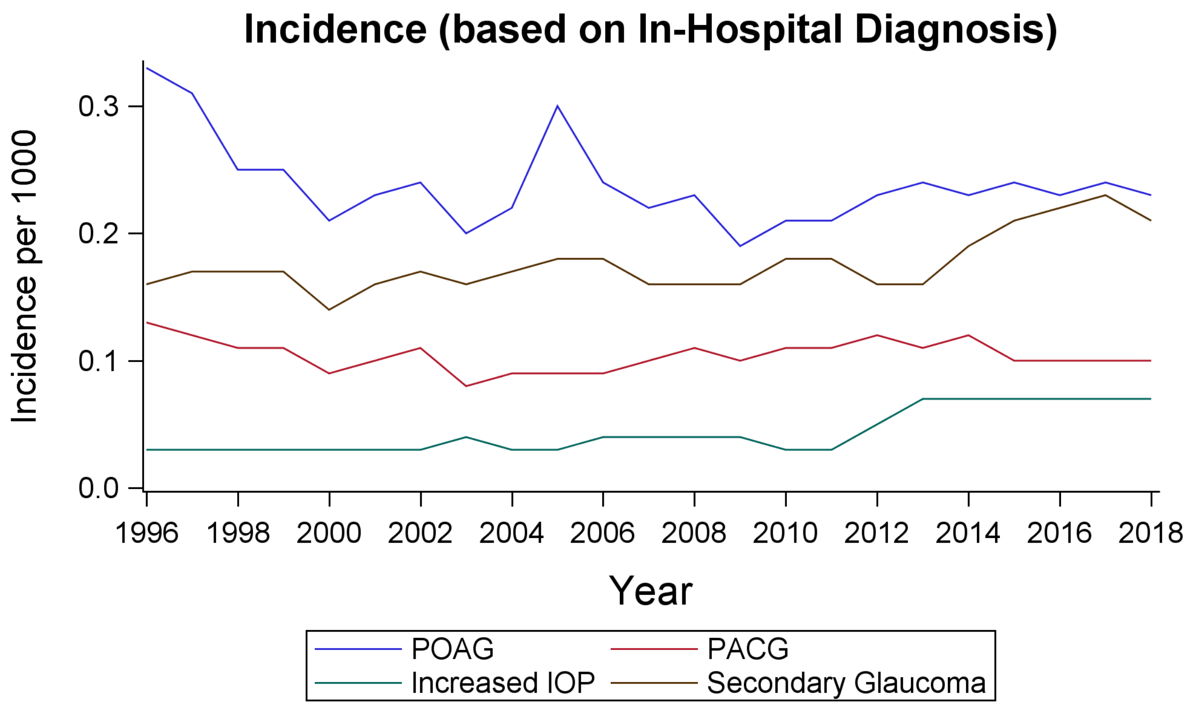

B

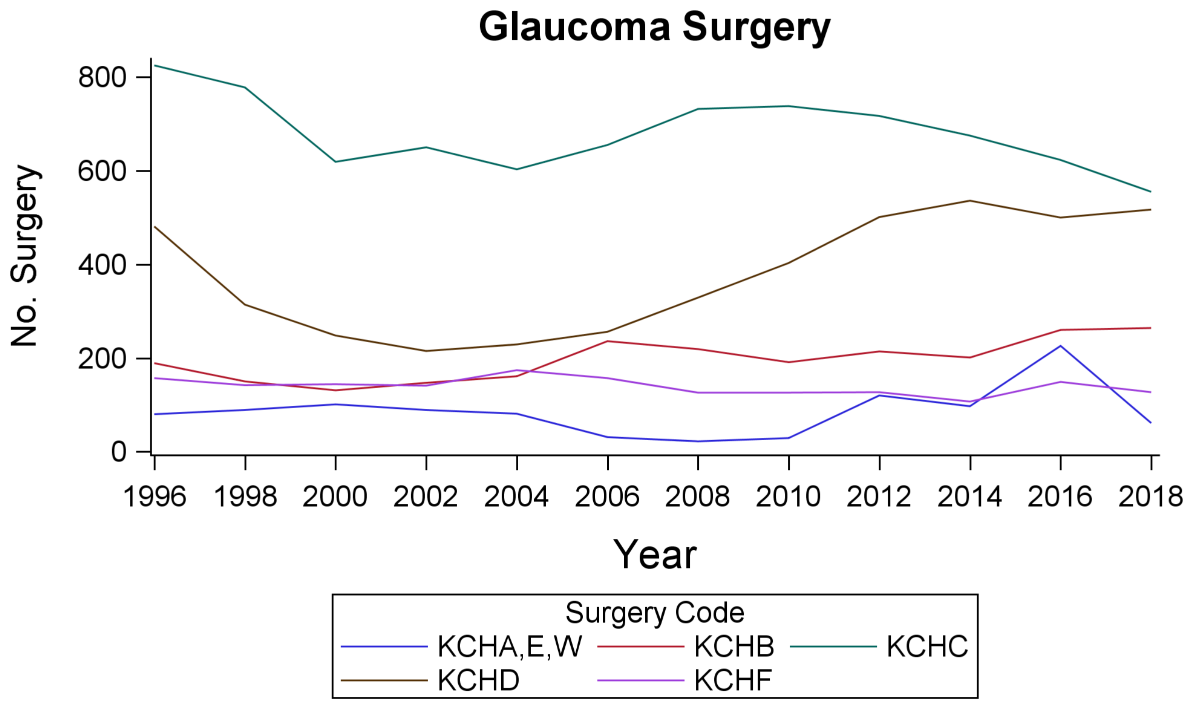

S2 Fig. The frequency over the period for the glaucoma-subtype measures, based on the diagnosis data and the number of operations. Panel A depicts the incidence of in-hospital ICD-10-diagnosed glaucoma cases in the 21-year period, divided into POAG, PACG, increased IOP, and secondary glaucoma. It reveals that POAG is the most frequent type of glaucoma in Denmark and that there exist similar trends in the prevalences of the different types of glaucoma. Panel B depicts the frequency of different IOP lowering operations in the 21-year period. Abbreviations are POAG: primary open-angle glaucoma; PACG: primary angle-closure glaucoma;

IOP: interocular pressure; KCHC: iridotomy; KCHB: surgery on first eye chamber and chamber angle (e.g., trabeculotomy); KCHD: Filtration operation; KCHF: ciliary body surgery.

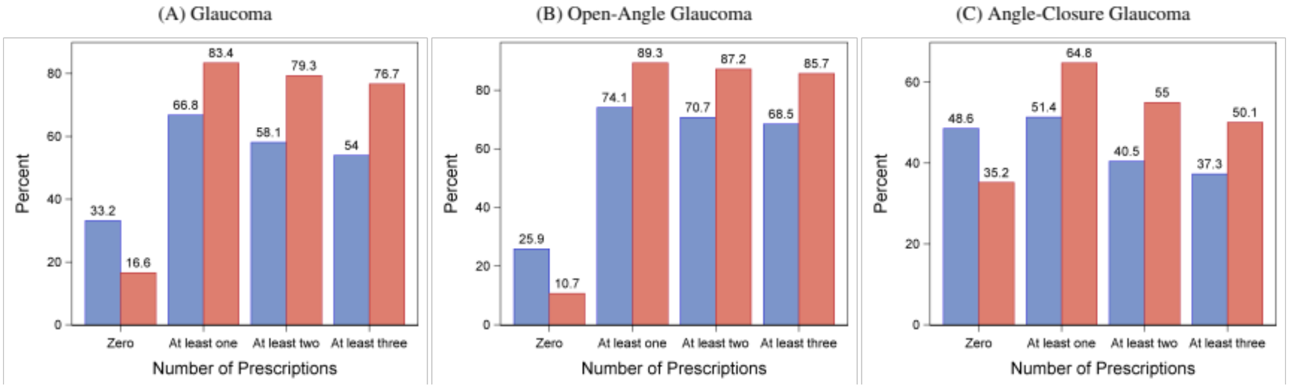

S3 Fig. Frequency of prescription counts in patients diagnosed with glaucoma. Blue bars indicate proportions for individuals with age≤40. Red bars indicate proportions for individuals with age>40 years. *Panel A*: Overall proportions of prescription counts in the combined sample of POAG and PACG diagnosed individuals. *Panel B*: Proportions of prescription counts in the sample of POAG-diagnosed individuals. *Panel C*: Proportions of prescription counts in the sample of PACG-diagnosed individuals.

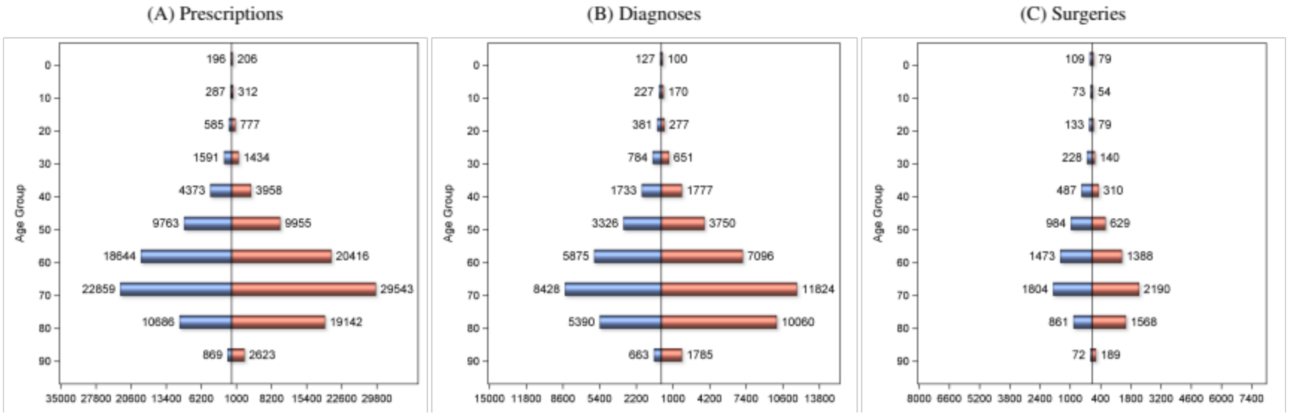

S4 Fig. Population pyramids. The population pyramids depicts the age distribution of individuals having reclaimed at least three anti-glaucomatous prescriptions, an in-hospital ICD-10-diagnosis or surgery. Blue bars represent males and red bars represents females.

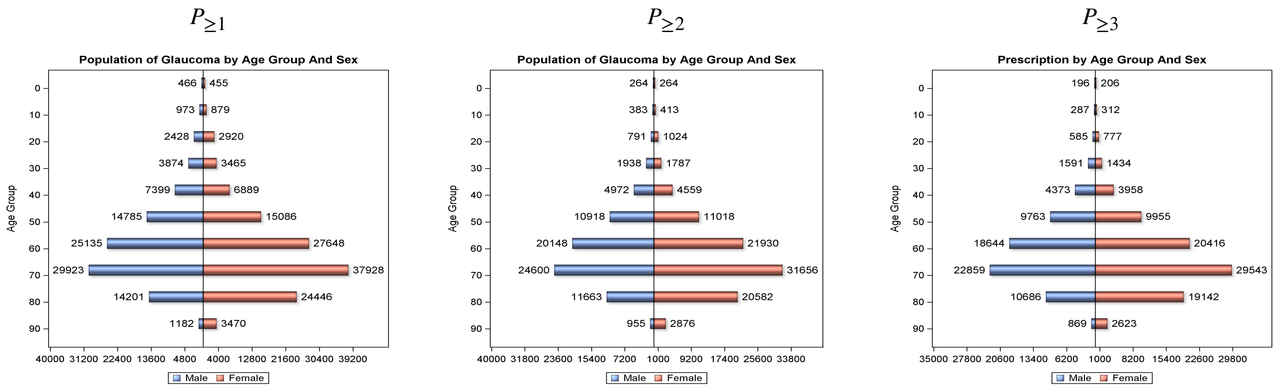

S5 Fig. Population pyramids for individuals having reclaimed at least one, two, or three anti-glaucomatous prescriptions.

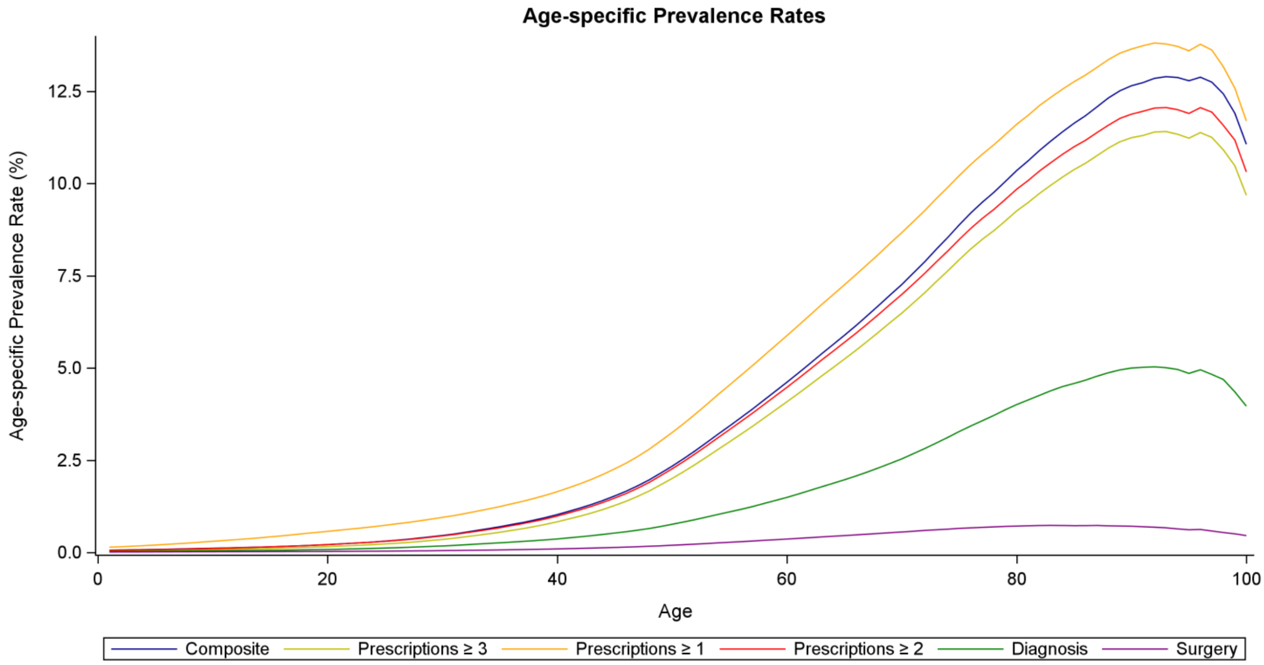

S6 Fig. Prevalence per age. The figure depicts the prevalence rate per 100 person-years (where glaucoma is observed in the 21-year period) shown for the claimed prescriptions sub-measures as well as the combined glaucoma measure.

S1 Table. Prescription-Based and Composite Measure Identification of Individuals

		Composite measure (G_c)		
		0	1	Total
**At least one prescription ($P_{\geq 1}$)**	0	6.687.388 (96.49%)	19.591 (0.28%)	6.706.979 (96.77%)
	1	59.298 (0.86%)	164.294 (2.37%)	223.592 (3.23%)
	Total	6.746.686 (97.35%)	183.885 (2.65%)	6.930.571 (100.00%)
	Jaccard index: 0.68			
		0	1	Total
**At least two prescriptions ($P_{\geq 2}$)**	0	6.734.314 (97.17%)	23.488 (0.34%)	6.757.802 (97.51%)
	1	12.372 (0.18%)	160.397 (2.31%)	172.769 (2.49%)
	Total	6.746.686 (97.35%)	183.885 (2.65%)	6.930.571 (100.00%)
	Jaccard index: 0.82			
		0	1	Total
**At least three prescriptions ($P_{\geq 3}$)**	0	6.746.686 (97.35%)	25.643 (0.37%)	6.772.329 (97.72%)
	1	0 (0.00%)	158.242 (2.28%)	158.242 (2.28%)
	Total	6.746.686 (97.35%)	183.885 (2.65%)	6.930.571 (100.00%)
	Jaccard index: 0.86			

Table note: two-by-two tables for the number of individuals identified by the composite measure (G_c) and the three prescription-based measures ($P_{\geq 1}$, $P_{\geq 2}$, and $P_{\geq 3}$).
